# Harlem Medical Association

**Published:** 1892-02

**Authors:** A. H. Leary

**Affiliations:** Secretary


### HARLEM MEDICAL ASSOCIATION.

Reported by A. H. LEARY, M. D., Secretary.

Stated meeting, December 2, 1891, Dr. M. C. O'Brien, President, in the chair.

Dr. G. H. Cocks presented a young man on whom he had successfully operated for tracoma. The lids were rolled over on the forceps and parallel incisions were made with a knife having three blades. A solution of bichloride, 1-500, was then employed to wash the lid and a stiff tooth-brush was also used to rub the solution well into the tissues. Dr. Cocks described another operation sometimes employed which does not require giving ether, being done only under cocaine. This consists of seizing the tracomatous tissue with forceps specially made for the purpose, having flat ends and pressing out the soft material ; then using the bichloride solution to wash the lid over the space operated on. A number of sittings are, however, required.

Dr. Fridenberg : The case presented is a very good result. These cases of tracoma must be kept from institutions for fear of contagion ; hence, any method of treatment which will insure a speedy recovery is useful so the patient may return to the institution. It is said that in Belgium alone there are some 4,000 cases of tracoma, the subjects being practically blind. I consider the treatment by scarification, as described in Dr. Cock's case, the best, as it requires but a few days' rest afterward and the patient can return to work. I have not been able to have the patient submit to the manipulation of squeezing and pressing with the forceps, even under cocaine. This latter plan of operation requires a long treatment and much waste of time. The radical treatment can be done by any practitioner.

Dr. T. H. Manley, in making some remarks on umbilical hernia, stated this condition is found nearly always in the female and mostly after labor. It has never been my experience to observe an inguinal or femoral hernia to result from labor. Recently I met a case of hernia in a man situated just above the umbilicus, which was said to have existed twenty years. Recently he had colicy pains and, two weeks ago, symptoms of strangulation. The tumor increased in size, and vomiting and constipation ensued ; but the vomited material did not become stercoracious. Under the hypodermics of morphia and hot applications the symptoms subsided without operation.

During the past year I was called to see a woman, sixty-five or seventy years of age, who was being treated by her physician for gastralgia. The vomiting took on a stercoracious character, and when I saw her she was in collapse. On examination an incarcerated umbilical hernia was found. The condition of the patient did not admit of operation, and a few hours later she died.

A woman recently came under my care at the Harlem Hospital suffering from umbilical hernia which, she stated, followed the birth of her sixth child. The tumor had gradually grown in size, and frequently gave symptoms of strangulation. Her physician had treated her with mild saline purgatives, with temporary relief, but the attacks became so frequent and painful that she desired operation. This case illustrated the fact that the hernial mass in the umbilical variety has no peritoneal covering, being covered by cellular tissue only. After cutting through the stretched and thinned integument, the gut and omentum were found in a pouch between the aponeurotic membrane and integument. The adhesions were very intimate. The stump of the omentum dropped into the abdominal cavity by the slipping off of the ligature, and a large amount of blood was lost. The incision was extended when three large blood-vessels were found requiring ligatures. These were easily secured. A favorable result followed the operation.

Dr. Manley then presented to the Association a colored woman on whom laparatomy had been performed some three years ago, for what exact condition she was unable to state. A large hernial tumor, the size of an adult head, occupied the situation between the umbilicus and the pubes in the median line. The incarcerating ring is small and the periods of strangulation are frequent and very painful. Hence, her urgent desire is for operation, which I expect to perform in a few days.

Dr. Manley further related a case of uterine fibroid, in which metrorrhagia was a distressing symptom. The attending physician had employed every known method of treatment, including electricity. This latter plan had been useful in numbing the pain, but had not reduced the size of the tumor. The patient was obliged to give up her situation and seek more radical treatment. I discovered a mass within the uterus the size of a cocoanut. All the organs were firmly bound down in the pelvis. While recognizing the seriousness of the operation of abdominal hysterectomy, I was about to advise this operation when I bore in mind a similar case coming under my observation some five years ago, in which the cervix was dilated and the mass was successfully removed by sections—a permanent cure resulting. Therefore, in this case, tents were used until two fingers could be introduced through the cervix, when some two tablespoonfuls of material were scraped off. This was repeated a number of times, and the vulsellum forceps were employed to remove one or two portions as large as an orange. A sanguineo-serous discharge continued for some time, but the uterus gradually assumed the normal size. Payon makes mention of 125 cases he has operated on with this method with only three deaths resulting from the operation. This does not, however, give any assurance of the ultimate result of the disease.

Dr. Wilbur F. Martin: I have recently had considerable success in treating two cases of uterine fibroid with electricity, employing a mild current of about 100 milliampères. The tumors are being gradually reduced in size.

Dr. Manley presented a specimen of the vertebral column taken from a young man who had fallen from a ladder at a height of seventy feet. No vertebral bones were broken, but there was extensive ecchymosis over the cervical region, due to concussion. The patient was paralysed below the shoulders, and felt as if a rope were tied around his body. After death a clot was found between the duramater and body of the vertebræ in the cervical region, and this produced fatal pressure on the spinal cord. The vessels of the cord and the cord itself were uninjured. I have seen this condition to exist in other cases, and I am confident this is the usual way a fatal result is produced, when the bones are not fractured, and the cord thus injured by the fragments.

Dr. Manley also exhibited a specimen of a popliteal artery, five days after being tied for an amputation at the knee-joint. The patient had received very extensive fractures and dislocations from being caught in revolving shafting, and died of septicemia. The clot in the specimen exhibited was firm and undergoing organization.

Dr. R. J. STANTON read a paper on

### A CASE OF SPINA BIFIDA OBSTRUCTING LABOR.

On the morning of August 23, 1891, I was called to attend Mrs. G. in confinement. She was twenty-six years of age. This was to be her first child, and she said that she was not expecting her labor for six weeks to come, and knew of no reason why it should be premature. She had been in excellent health all along, and, with the exception of edematous feet and legs, appeared to be in good condition. I was called at nine in the morning. She had been having pains since seven, having passed a very good night, sleeping until awakened by the pains. Upon examination I found the os pretty well dilated, but not entirely so ; high up I also found a pair of feet presenting. As the pains were weak and infrequent, I left, telling the nurse to send for me in case the pains grew stronger and increased in frequency, or in case a foot should come down. One hour afterward I was sent for, the husband telling me that a foot had been born. Upon my arrival I found the left foot and leg as far as the knee had been born. I had hardly time to get my coat off when the mother complained of a peculiar feeling coming over her. Her face gradually blanched, she grew restless, throwing her hands and arms about, yawned, grew thirsty, the pulse at the wrist grew more and more feeble, until it was scarcely to be felt at all. In a word, she had all the signs of collapse, and it alarmed me, as I saw absolutely no cause for such a condition. I resolved to deliver her as rapidly as possible. I brought down the other leg, and with steady traction endeavored to extract the trunk. It came with increasing difficulty until the breech was passed, but further traction caused the mother such terrible agony that I saw that some obstruction existed somewhere. Passing my fingers under the child's back and between it and the posterior vaginal wall of the mother, they came in contact with something giving precisely the impression of a tense bag of waters. I then found that the more traction was exerted upon the child the lower and firmer did this bag of waters, or whatever it might prove to be, wedge itself between the mother and child, thus very effectually preventing the delivery of the latter and causing excruciating pain to the former. In the meantime the mother seemed to be in a very precarious condition. At times she would have severe chills, and at other times would lie in a semi-comatose state.

I plied her with brandy, digitalis, and other stimulants, and after a while she rallied. I was of the opinion that it was a case of concealed hemorrhage, and that the fluid contained in the tense tumor or sac was blood, but what it was that formed the walls of the sac I was at a loss to determine. At this stage of the proceedings I thought it well to have the opinion in the case of someone more skilled than I, and, accordingly, my friend, Dr. James A. J. O'Brien, was sent for and shortly after arrived. He examined and, while not agreeing with my diagnosis, conceded that it was a case which easily admitted a difference of opinion. He thought that it might possibly be a monstrosity of some sort, which in the end proved to be nearer to the truth than did my idea. However, whatever the diagnosis, the treatment was clear enough, which was to rupture the sac and complete the delivery as soon as possible. I first inserted a hypodermic needle up into the sac and withdrew a syringeful of a straw-colored fluid, proving that my idea, at any rate, was not the correct one. The sac was then ruptured, and fluid gushed out to the extent of almost filling a quart vessel. After the sac had been emptied, the rest of the delivery was easily enough accomplished. The placenta showing no disposition to follow, was extracted with the hand, proving to be a very small one, not more than one-half the usual size and somewhat fatty, besides appearing torn and macerated, although it had been not adherent, and no violence had been done while extracting it. The hemorrhage was very slight, not more than in an ordinary case. The uterus contracted well and the woman went on to recovery without any complications of any kind.

Upon examining the fetus, the cause of the obstruction to the delivery was at once apparent. The child, a female, was dead and evidently had been so for some days. Its weight was about seven pounds, but it appeared to be a full term child. The parietal bones seemed to be somewhat abnormally developed in size, while the occipital bone was entirely wanting. Over the space left by the absence of the occipital bone was a spina bifida sac, its walls composed of a continuation of the scalp shading downwards into the integument of the neck, and the sac extending downwards to the fifth or sixth cervical vertebra, being attached to the spinal column to that extent. Its attachment was constricted like a neck, while the sac itself was round like a cocoanut and about the same size—that is, somewhat larger than the child's head. The child seemed to be normally developed otherwise, with the exception of the lower jaw, which was retreating, and did not seem to have reached its full development, while inside of the mouth the jaws, instead of being covered by the gums, were of a light colored cartilage, giving the child the appearance of having a mouthful of teeth. The spina bifida sac, of course, explained the difficulty in the delivery, but what it was that caused the almost total collapse of the mother I am still at a loss to explain. It may have been due to fright, pure and simple, as the nurse was a novice, and upon seeing a foot appear was very much alarmed, not knowing what to think, and this alarm may, in a measure, have been shared by the young mother. I know of no other plausible explanation.

My reason for reporting this case is very simple. To me it was an exceedingly interesting one, and extremely profitable as well. Cases of spina bifida obstructing labor have been reported before, but they are still so exceedingly rare, that when the obstetrician is confronted by such a case, he is not always apt to think of such a complication, and, therefore, it is well that such cases be reported from time to time, so that the possibility of such complications may be kept well in mind. Therefore, I report the present case with the hope that it may prove of value to others as it has to me.

Dr. A. H. Leary related a case of spinal bifida which very recently came under his observation, where the covering of the tumor, which was as large as an orange, exuded blood. The integument was as thin as tissue paper. It collapsed the seventh day after birth, and the infant was found dead. No convulsions were noticed. The previous history of the mother was very bad, she having been treated some five years before with very extensive scrofulous abscesses.

Dr. Malleson described a case coming under his notice, in which the vertebræ were mostly composed of cartilage, there being only a few bony centers. It was learned that the mother suffered from chronic peritonitis and very frequent attacks of rheumatism. The family record was bad as far back as could be learned.

Dr. Stanton stated he did not know the previous history of his case, as he had only attended the patient during the time of her delivery.

Dr. O'Brien had seen three cases of spinal bifida, and in all there was a tubercular history.

Dr. A. Chevalier read a paper on

### A CASE OF PERNICIOUS ANEMIA.

I was called, on the 22d of last March, to see Mr. W. He gave the history of being fifty-four years old, an Englishman by birth, a salesman by occupation. He drank ale in moderation, but otherwise his habits were temperate. His family history was good, each of his parents being over seventy years of age, and still healthy.

He had never required the services of a physician before, and had always regarded himself as perfectly healthy until about three weeks before he sent for me. He then began to notice that he was becoming very pale, and that he was more easily fatigued than usual, and that he had difficulty in going up stairs, having to stop frequently on account of dyspnea and palpitation. The symptoms had gradually grown worse, and he had fallen from an attack of vertigo while going up one flight of stairs on the night that I first saw him.

On examination I found him to be a well-proportioned man, of 170

pounds weight, five feet nine inches tall. The most remarkable thing in his appearance was his extreme paleness, his lips, tongue, and conjunctivæ being almost bloodless, and his skin was of a pale straw color. His temperature was 100½, his pulse full, soft and regular, beating about 104 a minute while he was lying down.

There was marked pulsation of the carotids, and a loud anemic murmur could be heard over those blood-vessels. It could be heard also at the base of the heart and with less intensity at the apex. His heart seemed normal in size and location. His lungs, liver, and spleen also seemed to be normal. His urine was light amber in color, sp. g. 1014, acid, and at this time contained no albumin. Up to this time his appetite had been good, and his bowels were regular.

There was no edema, petechia spots, or retinal hemorrhage, although the optic disc was much paler than in health. In order to get enough blood to moisten a slide for examination, it was necessary to make a long incision into the finger and then use pressure toward the wound. The blood was light in color, leaving a lemon-colored stain on a piece of white linen. On examination the red corpuscles were very much reduced in numbers. In shape they were irregular, and in size they varied from being twice as large as normal to others not more than one-third to one-half the usual size of a red corpuscle.

The white corpuscles showed nothing unusual. They seemed slightly increased as compared to the red, but for the mass of the blood there was probably a slight diminution.

The patient was put under treatment, and for a period of about two weeks he became steadily worse. His temperature varying from 100 to 103, his pulse beating about 120, slight edema of the feet and legs, and more marked prostration, so that he was obliged to keep his bed most of the time. During this time he suffered from anorexia, but no nausea or distress after eating, and his bowels continued to move but once a day. He now had three attacks of epistaxis, in each of which there was slight oozing of blood for from six to twelve hours. On the 5th of April his appetite began to return, the slight edemia disappeared, his fever gradually lowered until the last of April, when it became normal, his pulse now beating about eighty a minute, and he expressed himself as feeling strong, and determined to return to work, which he did on the first of May, although his complexion, blood and anemic murmurs remained as when I first saw him, and he complained of dyspnea when going up stairs the length of those at the 125th Street and 8th Avenue elevated station. During May he was at business from eight to five each day and felt fairly good, but at the beginning of June his anorexia and weakness returned, and he was obliged to keep his room.

Now for a period of two weeks he passed out from under my care.

When he sent for me again, on the 16th of June, I found him very much prostrated. His feet and legs were very much swollen, his fever was 102, and his urine now contained about ten per cent. of albumin.

He was at times delirious and his appetite was entirely gone. He remained in this condition for about one week, when a severe diarrhea occurred. The first of his sickness came on, the edemia disappeared, and his mind became clear. At this time he had two more attacks of epistaxis. His temperature fell to ninety-seven, and he expressed himself as feeling much better. His appetite, however, did not return, and his diarrhea continuing, he died of asthenia on the 12th of July. A post-mortem was not allowed.

The principal features of the case that have interested me was, first, the etiology. It is quite common under the age of forty, especially in females, where it may follow parturition or exhausting discharges. It also frequently follows stomach or intestinal disorders and tape-worm. It is also frequently seen in insane asylums, where it follows mental worry, but in this case it must be regarded as purely idiopathic in a man of fifty-four of a sanguine happy disposition.

Another point of interest was the slight amount of alimentary disturbance during the course of the disease. He never suffered from nausea or vomiting. Anorexia was not marked until the last three weeks, and there was no diarrhea until the last seventeen days.

Another point was the period of remission of the disease. I think if I had more strongly urged rest during the month of May, instead of allowing him to return to work, that his life might have been prolonged several months, although the final ending of the disease would probably have been the same.

And, lastly, the slight amount of hemorrhage. He had epistaxis five times, but there were no ecchymotic spots or retinal hemorrhages.

Dr. Martin related a case which had recently went the rounds of the hospitals. Pernicious anemia had been diagnosticated and treated accordingly, but with what success he could not state, as he had recently lost sight of the patient. A very interesting fact connected with this case was that there was a universal alopecia. Scarcely a hair could be found over the whole body.

---

It is to be deplored that so many excellent medical journals offend good taste by publishing interleaved advertisements.

